# Unmotherly

**Published:** 1870-06

**Authors:** 


### Editorial.

### UNMOTHERLY.

"Aromatic ledge of rocks,
O'erhung with wildwoods' thickening green,'

Was bountifully bedecked with clusters of hanging fruit, whose ruddy ripeness ravished the senses of sight and smell, as a group of longing hungry children cast wishful glances at the glowing berries. No subtile serpent was needed to persuade them that here was fruit "good for food," if not "desirous to make one wise." But practically it was to them "forbidden fruit," for it was beyond their reach. An adventurous, benevolent boy resolved to scale the cliff, and furnish his companions the coveted clusters. Having reached a secure position on a projecting rock, within reach of the objects sought, he had just begun to hand down the delicious fruits, receiving as a reward the smiles and cheers of his little playmates, when he was seized by the foot and rudely jerked from his position.

Doomed to disappointment, while fully conscious of the unselfishness of his motive and action, the brave boy promptly resented the rudeness. But, finding that the hand which had so unfeelingly arrested his generous career was his own mothers', he silently stole away, hoping his comrades would not discover the relationship.

If not that, good reader, then this:

Only a few years ago, humanity was suffering untold agonies which none were trying to relieve. Unweaned babes were tortured with toothache. Toothache!

"Horrid! wild! oh! murderous pain!"

Something that made even the turnkey welcome! Then the "maiden's smile" was a ghastly grin, bestudded with snaggled ghosts of departed dentures; her balmy breath was a barn-yard stench, and even her bridal kiss was but a poultice of pollution! A prattling babe shrinking with disgust from the proffered kiss of its fond, but foul-mouthed mother! "Ponder it! Think of it!"

And did *general* surgery remain idle, in view of all this misery? Certainly not! She helped the blacksmith to use the turnkey; and into the cup of oral woe, already full to the brim, she poured the flood of mercurial ptyalism.

And all this time the needed relief hung, in beautiful clusters of fruitful knowledge, but a little way up the side of the hill of science.

The spirit of *Dental Surgery*, like the generous boy, resolved to climb up and hand it down. The better to facilitate his labor, he constructed scaffoldings of Dental Societies, Dental Colleges, Dental Periodicals and Dental Text-books; and when just properly organized for work, how must he feel to find that his own mother is trying to pull down his scaffolding, while he is on it, and humanity under it?

We have been led to the above reflections in view of a fact that in an enlightened city of the central West, a Medical College, with ten or more professors, has also a "Lecturer on the Elements of Dentistry," and proposes, on the strength of this, to grant suitable diplomas to those who wish to graduate only in Dentistry. Just think of this wonderful institution, with fifteen teachers, in a session of only five months, making full-fledged Dentists, solely through the influence of one subordinate! Suppose one of the Dental Colleges should have a physician give five or six lectures on the Elements of Medicine, and then grant the degree of M. D. to those who prefer it to D.D.S. Pshaw! we feel worse than waterbrash.

Lectures on Dental Surgery in a Medical College, if properly prepared and delivered, will prove highly useful to physicians, as many of them do not know which permanent tooth is first developed, nor where it makes its appearance. But when physicians deliberately propose to make Dentists as above, they deserve to be ptyalized with their own mercury, and have their teeth extracted with their own turnkeys, were it not that like Paul with his persecutions, they do it "ignorantly in unbelief." W.

